# Status, challenges and facilitators of consumer involvement in Australian health and medical research

**DOI:** 10.1186/1478-4505-8-34

**Published:** 2010-11-18

**Authors:** Carla Saunders, Afaf Girgis

**Affiliations:** 1 Centre for Health Research & Psycho-oncology (CHeRP), Cancer Council NSW, The University of Newcastle & Hunter Medical Research Institute, Callaghan NSW, Australia, 2308

## Abstract

**Background:**

The emergent international practice of involving consumers in health research is driven, in part, by the growing share of health research that can only be applied in and emerge from knowledge that is shaped by human values and societal contexts. This is the first investigation of its kind to identify the current prevalence, challenges, enabling factors and range of approaches to consumer involvement in health and medical research in Australia.

**Methods:**

A nation-wide survey of research funding organisations and organisations that conduct research was performed during 2008-2009.

**Results:**

Marked variation in consumer involvement experience and perceptions exists between research funders and researchers. Research funders were over eight times more likely than organisations conducting research to involve consumers in identifying research needs and prioritising research topics. Across both groups, practical and time constraints were reported as key challenges to involving consumers, while guidelines on consumer involvement and evidence of effect were the most important potential enablers. More than a third of research organisations indicated that when consumer involvement was a condition of research funding, it was an important facilitator of involvement.

**Conclusion:**

It is no longer simply enough to keep society informed of important scientific breakthroughs. If Australian health research is to take into account important social contexts and consequences, it must involve consumers. A set of minimum consumer involvement standards and associated guidelines, that are agreed and routinely adopted, could ensure that consumers and the Australian community they represent, are given an opportunity to shed light on experiences and local circumstance, and express views and concerns relevant to health research.

## Background

Health and medical research is scientific investigation performed to better understand human health and wellbeing [1]. Its goals are concerned with protecting and improving health and quality of life; and it utilises methods across the spectrum of investigations from biomedical inquiry through to examining human behaviours [2]. Advances resulting from health and medical research are the basis for improved health standards and increased longevity, and are a primary driver of innovation and increased productivity [3]. Justification for universal progress in health science is motivated by the need to address the rising prevalence of chronic non-communicable disease worldwide [4].

The effects of health science research on society present a mixed record of benefit and harm. There are cases when health and medical research has led to increased mortality and long-term suffering and hardship, such as the exposure to many millions of people worldwide to diethylstilboestrol, an oestrogenic medicine discovered to have harmful long-term health consequences in three generations to date [5]. There are also 'grey areas' where there is potential for benefit, but also conflicts of interest with ethical or moral views, such as embryonic stem cell use. It is argued that since scientists cannot necessarily foresee the social effects of research or make value based or contextual decisions about them alone, decisions about health science should routinely be made in discussions with the non-scientific community [6].

The importance of better connecting health and medical research with society has been emphasised for several decades [7-9]. It is argued that, as health and medical research is a social process, it should be informed by the interactions of researchers and potential end beneficiaries, where both groups exchange experiences, ideas, views and expectations and combine this knowledge into acceptable, realistic research objectives [10]. Consumer involvement is believed to reduce opposition to implementation of the research [11]; build commitment to the research findings and needed organisational changes [12]; enhance the quality of the research design by improving the overall understanding of the solution [13]; help avoid development of unacceptable health interventions; and provide necessary expertise about the community processes that will be supported by the research findings [14].

Assessments of existing levels and types of consumer involvement have been made by several groups overseas [15,16]. Overall, they found a considerable proportion of researchers had experience working with consumers and that involving them required appropriate skill, resources and time to develop and follow appropriate protocols [15]. This investigation sought to improve current knowledge of consumer involvement in health and medical research in Australia through an assessment of the prevalence of, challenges with and facilitators to consumer involvement in research and research funding organisations.

## Method

This study was conducted over a 12-month period beginning in August 2008; with ethics approval from the University of Newcastle's Human Research Ethics Committee.

### Participants & sample size

Research and research funding organisations were surveyed to gain a broad perspective on consumer involvement from both a governance and functional position. The multiple stakeholder approach was adopted to reduce potential bias from measuring consumer involvement from a single-group perspective.

The study design featured relatively broad inclusion criteria including all health and medical research organisations conducting non-commercial human and biomedical research such as universities, independent medical research institutes and health service research units. Non-government research funding organisations such as health charities that financially supported this type of research were also included. Exclusion criteria included commercial research groups; government agencies; those that examined existing or proposed health policy and international health research organisations. Health charities without an external research funding program were also excluded.

*Research funders *were identified from directories available from ProBono Australia [17], Auscharity [18] and Philanthropy Australia [19]. *Research organisations *were indentified from listings from NSW Office of Science and Medical Research [20], National Health and Medical Research Council [21], Universities Australia [22] and the Australian Government Directory 14^th ^Edition [23]. A search of individual websites was also undertaken of Australian health charities, universities, independent medical research institutes and health service research units to assess and/or confirm eligibility of identified health research funders and organisations and to identify additional organisations for inclusion. Three hundred and six (306) organisations were identified as eligible; 89 research funders and 217 research organisations.

### Survey instrument

The survey instrument was based on the international literature on involving consumers in health research [24,25]; and included eight primary items. Practical aspects and the degree and nature of current organisational commitment for consumer involvement were examined. Participants selected responses from a list of 13 areas known to engage consumers and nominated additional areas of involvement (Table 1). The availability of organisational resources, staff and protocols for involving consumers were also investigated in three subsequent *'Yes/No' *questions. Study participants were then asked to prioritise a list of seven challenges of involving consumers and submit other challenges they may have experienced (Table 2). For the final item, participants were asked to rate eight potential enabling factors they believed would be most important in increasing their organisations capacity to involve consumers or initiate consumer involvement in research. They could also provide descriptions of other possible enablers.

**Table 1:** Current involvement of consumers in research

Current involvement	Research funding organisation		Research organisation		Total	
	**Freq**	**%**	**Freq**	**%**	**Freq**	**%**

Input into organisational governance (organisation wide committee member)	44	60	72	75	116	65
Fundraising	33	57	45	38	78	44
Disseminate research information	33	57	45	38	78	44
Individual research project committee member	22	38	30	25	52	29
Identify research needs	35	60	8	7	43	24
Prioritise research	35	60	8	7	43	24
Input into acceptability of proposed research and likelihood of participation	14	24	25	21	39	22
Recruit participants	5	9	28	23	33	19
Other (ethics committee member)	0	0	28	23	28	16
Assist the development of research funding applications	8	14	5	4	13	7
Assist the development of research tools e.g. participant surveys or information sheets	3	5	8	7	11	6
Gather/facilitate research data collection	4	7	7	6	11	6
Member of research grant review panel	9	15	0	0	9	5
Contribute to the formulation of research policy such as funding guidelines	8	14	0	0	8	4
Other (felt laboratory research was not applicable)	0	0	8	7	8	4
Other (planning to in future)	2	3	2	2	4	2
Other (provide community talks)	0	0	2	2	2	1

**Table 2:** Challenges to involving consumers in research

Challenges	Research funding organisation		Research organisation		Total	
	**Freq**	**%**	**Freq**	**%**	**Freq**	**%**

Practical and time constraints (developing lay information, consumer policies, office space, time to orientate etc)	40	69	46	39	86	49
Clarification of consumer roles and responsibilities	25	43	40	34	65	37
Accessing consumers	27	46	31	26	58	33
Cost (reimbursement of out of pocket and other costs such as new office equipment etc)	20	34	1	1	21	12
Negative or complacent attitude of paid staff toward consumer involvement	7	12	14	12	21	12
Feedback requirements of consumers (recognition and appreciation requests)	13	22	7	6	20	11
Difficulty working with consumers (accepting opposing views, outspoken views etc)	5	9	9	7	14	8

### Procedure

An information statement and survey were initially sent by email to the Chief Executive Officer (CEO), Director or a senior researcher of the 306 identified health research and funding organisations. The email recipient was asked to forward the request for study participation to someone more suitable to complete the survey on behalf of the organisation, if required.

### Analysis

Quantitative data were analysed using SPSS version 16 for Windows [26] and are presented as frequencies, percentages and cross tabulations. Open ended responses were examined to identify themes, and then individual responses were classified according to these themes.

## Results

### Response rate

As all primary databases of Australian research and research funding organisations were interrogated, along with extensive general searches, we are confident that the large majority if not all eligible organisations were identified and invited to participate. Responses were received from 177, (58%) of the 306 invited organisations; representing 65% (n = 58) of the research funders and 55% (n = 119) of the research organisations invited to participate. The majority of individuals (88%) who completed the survey on behalf of their organisation were those originally contacted. These individuals were considered most likely to have an understanding of consumer involvement in research in their organisations i.e. the CEO, Director or a senior researcher. The majority were male (67%) and aged between 40 and 60 years of age (88%). Most organisations had been established for at least 10 years and had more than 10 employees.

### Current consumer involvement

Participants were asked how their organisation currently involved consumers (Table 1). The most frequently reported involvement across both groups were: input into organisational governance, such as membership on strategic research planning or other high level committees such as the Board of Directors; fund-raising for research; and assisting with the communication and dissemination of information about individual or organisation-wide research activities. More research funders (57%) than research organisations (40%) involved consumers in fund raising for research and disseminating information about research. Across both groups, consumers were least likely to be involved in research policy development, research data collection and research grant review.

Overall, research funders were more likely to currently involve consumers in research processes and structures. They were also over eight times more likely than organisations conducting research to involve consumers in identifying research needs and prioritising research topics; whilst organisations conducting research were more than twice as likely to involve consumers in recruiting research participants.

### Organisational commitment

Overall, 15% (n = 25) of respondents reported that resources were specifically allocated to consumer involvement and 10% (n = 17) had a designated person responsible for facilitating consumer involvement in research. The availability of policies and/or protocols that require consumer involvement was reported by 43% (n = 51) of research organisations, who commonly reported Ethics Committee Terms of Reference, compared with 29% (n = 17) of research funders.

### Challenges to involving consumers

Almost half (49%, n = 86) of all respondents reported practical and time constraints (e.g. developing lay information and/or consumer policies, providing office space, time to orientate and communicate etc) as the most important challenges to involving consumers, with a higher percentage of research funders reporting this challenge (69% compared to 39% of research organisations). As indicated in Table 2, other frequently reported challenges include clarification of consumer roles and responsibilities; and the ability to access consumers. The perceived costs of involving consumers and the feedback requirements of consumers were reported as a challenge by 34% and 22% of research funders, respectively, with minimal reporting of these challenges by the participating research organisations.

### Perceived facilitators of consumer involvement

Sixty percent (n = 107) of all respondents selected guidelines and other practical information and tools to support consumer involvement as the most important potential aid to involving consumers. A close second was the need for evidence of organisational benefit for involving consumers (58%, n = 104), which was reported by a greater percentage of research organisations (65%, n = 77) than research funders (46%, n = 27). Third was the need for staff training to support consumer involvement (39%, n = 69).

More than a third of research organisations indicated that when consumer involvement was a condition of research funding, it was an important facilitator of involvement. Having an organisational driver (staff member in management with the necessary passion, skill and commitment toward consumer involvement) was more important to research organisations than to research funders. Of least importance as an enabler to consumer involvement across both groups was endorsement of the practice from other research organisations.

## Limitations

The response rate of 58% does not reflect the practices of over 40% of the initial sample, with possible systematic differences between respondents and non-respondents in terms of the nature and level of consumer involvement.

The study may have principally attracted responses from organisations who wished to showcase their efforts in involving consumers in research and may, therefore, reflect a higher level of consumer involvement than is actually practised.

Although key people, such as CEOs and other senior staff, were targeted in this research, these single organisational participants may not have been aware of all on-site consumer involvement practices, especially those from large organisations.

## Discussion

A study examining consumer involvement in Australian health and medical research is overdue. To our knowledge, this is the first investigation to identify the current level, challenges, enabling factors and range of approaches to consumer involvement in health and medical research in Australia. There is, in theory, wide acceptance of the potential benefits of involving consumers in health and medical research in Australia [27], but equally, in the practice of some groups, this study has found a reluctance to test these potential benefits.

In the context of a growing array of new and increasingly complex health care interventions that require public acceptance and uptake, the current involvement of consumers in Australian health and medical research can perhaps best be described as ambivalent. Consumer participation via committee membership such as an ethics committee was reported by 75% of the research organisations. On face value this may appear to be a high level of involvement however this cursory approach is unlikely to provide a genuine opportunity for consumers to influence health research in Australia. In 1969 Arnstein [28] simplified citizen participation using an eight-step ladder of hierarchical participation that remains in common use today. When participation is restricted to the low rungs of the ladder it signifies 'tokenism' where any effect is unlikely.

The breadth of literature on this topic indicates there is no single phase of research or research governance that consumer involvement is best suited. Nor is there a limit on the level of involvement that could be pursued [24,29,30]. Consumer involvement includes almost any kind of engagement between the researcher and consumer including in research prioritisation processes, at the design, planning, implementation and information dissemination stages of a research project, and in research governance and policy [31].

A UK investigation has found comparable challenges related to consumer involvement in research, including lack of time and other practical and resource constraints. They also found a similar reluctance on the part of basic biomedical researchers to accept consumer involvement in research as being relevant to them [32]. Yet the involvement of consumers has contributed to the relevance and quality of basic biomedical research including the identification of previously unstudied problems such as comorbid chronic disease [33].

Other research on this topic has found that involving consumers presents a change in the usual practice of researchers [34]. However our findings, of the apparent willingness of some research organisations to broadly involve consumers, suggests that greater cooperation between the two might be improved by addressing the identified challenges, including the need to formally investigate the advantages of involving consumers. Additionally, more Australian researchers may need to be aware of the potential for involving consumers alongside other members of a multidisciplinary research team with a diverse range of skills, knowledge and expertise. This is well accepted in the UK where the role of consumers in research is given a high profile and allows consumers to be recognised as genuine partners in research decision making processes [35].

This study identified the most important perceived facilitators of consumer involvement in research to be practical tools that advise and assist involvement. Australia essentially lacks a range of structures and mechanisms found to be useful by other countries in supporting consumer involvement. One example is the INVOLVE organisation which is funded by the UK Department of Health. INVOLVE was established to promote consumer involvement in research and improve the way that research is prioritised, commissioned, undertaken, communicated and used. The organisation provides resources and training for researchers and funders on how to involve members of the public. It also supports consumers who are thinking about getting involved in research. Through its website and publications it works with others to continuously build knowledge and understanding of consumer involvement [35]. The availability of effective resources and training programs, that facilitate the process of consumer participation, have been found to be important determinants of the benefits derived from involvement [36].

## Conclusion

The part played by science in society is becoming more influential [37]. A national model framework for consumer involvement in health research exists, on paper, in Australia [27]. However its usefulness is diluted by a number of structural and strategic factors including a lack of resourcing; a failure to properly embed consumer involvement into strategic research objectives and funding and review processes; an absence of mechanisms to fully support involvement; failure to link consumer involvement to other strategic goals and unclear responsibility for implementing consumer involvement among key stakeholders.

As a considerable proportion of new scientific knowledge can only be applied in and emerge from processes that are underpinned by human values and societal contexts, and given the heightened political importance of reducing health care costs, it may be timely to give an increasing focus and commitment to consumer involvement in health and medical research in Australia.

## Competing interests

The authors declare that they have no competing interests.

## Authors' contributions

CS is the first author of this paper. CS and AG conceived the paper. CS undertook the survey; data analysis and literature review. AG supervised the project design, implementation and analysis and contributed to the development, drafting and editing of the paper. All authors have read and approved the final manuscript.
